# Supplementary material for: Structural basis for late maturation steps of mitochondrial respiratory chain complex IV within the human respirasome
Source: Nat Commun. 2026 Jan 10;17:1550. doi: 10.1038/s41467-025-68274-3 (PMC12894743; doi:10.1038/s41467-025-68274-3)
Supplement: Supplementary file 1 — Supplementary Information [file 41467_2025_68274_MOESM1_ESM.pdf]

Supplementary information

Structural basis for late maturation steps of mitochondrial respiratory chain complex IV within the human respirasome

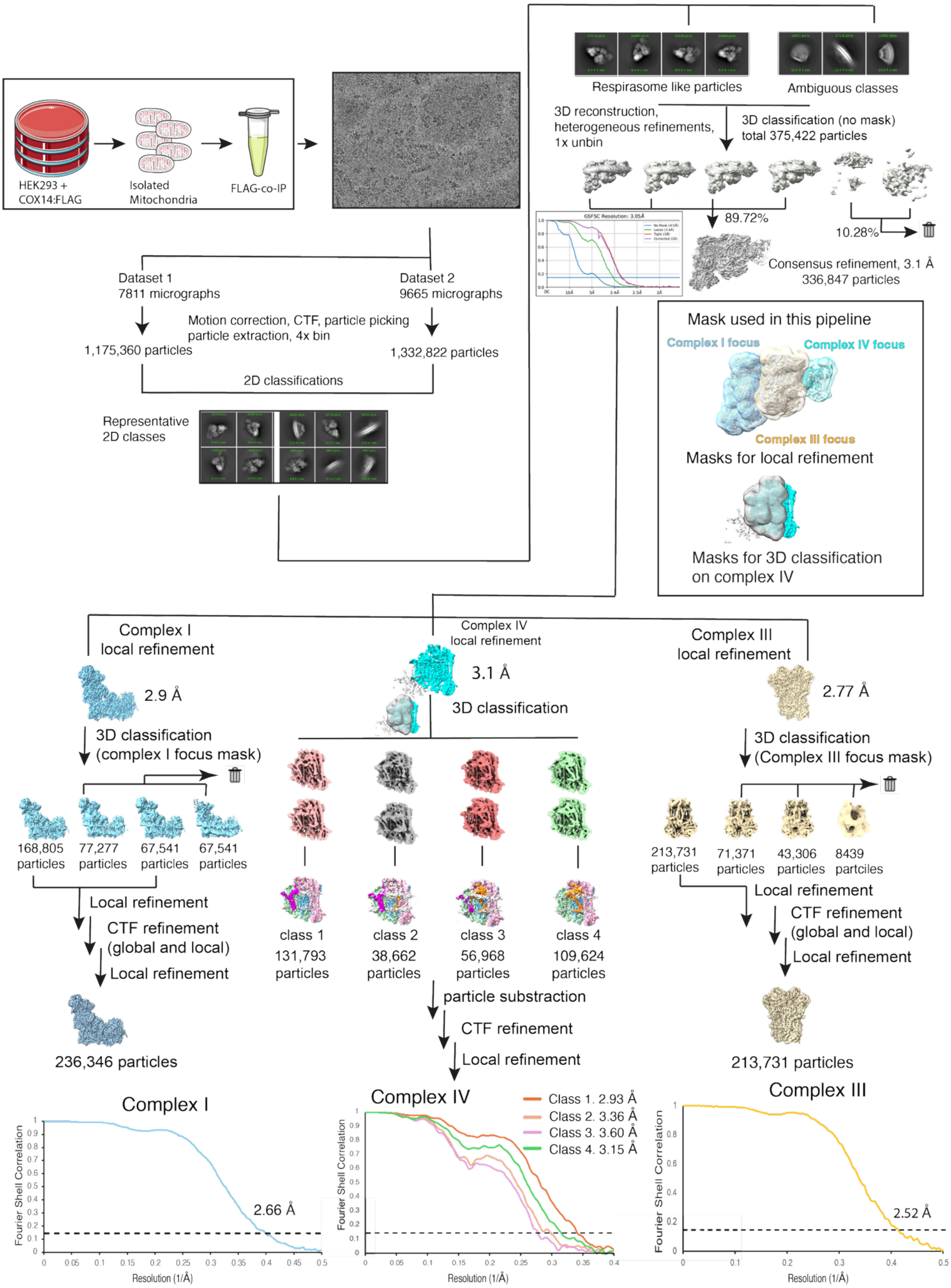

Supplementary Figure 1. Cryo-EM data collection and data processing
Data processing strategy and the Gold-standard Fourier shell correlation (FSC) for the EMDB-deposited reconstructions. Images in the top-left inset were adapted from Servier Medical Art, licensed under CC BY 4.0.

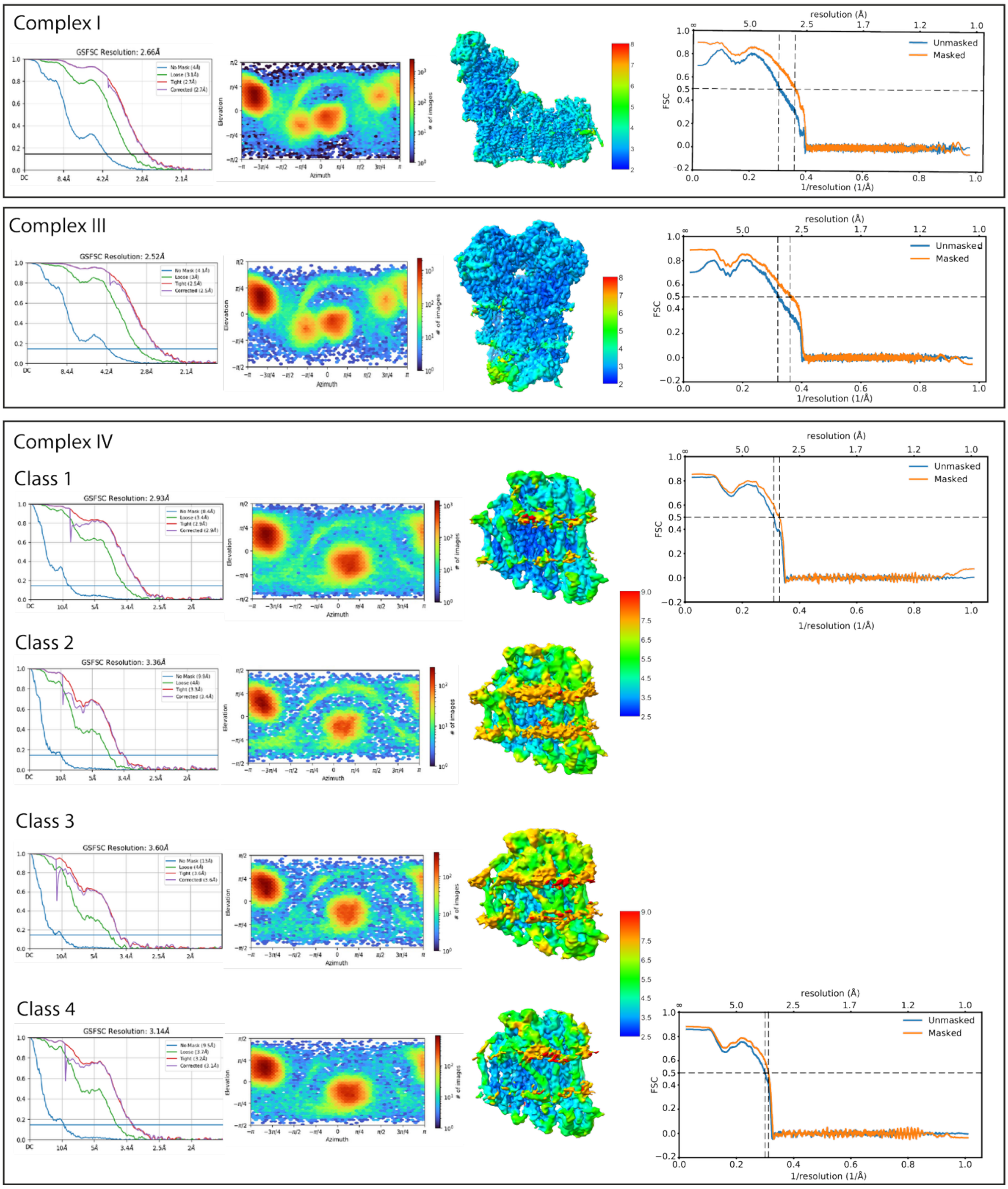

Supplementary Figure 2. Data processing and map validation
Gold standard Fourier shell correlation (GSFSC) plots, angular distribution plots, local resolution distribution for all maps, and map-to-model FSC (unmasked and masked) curves for four models.

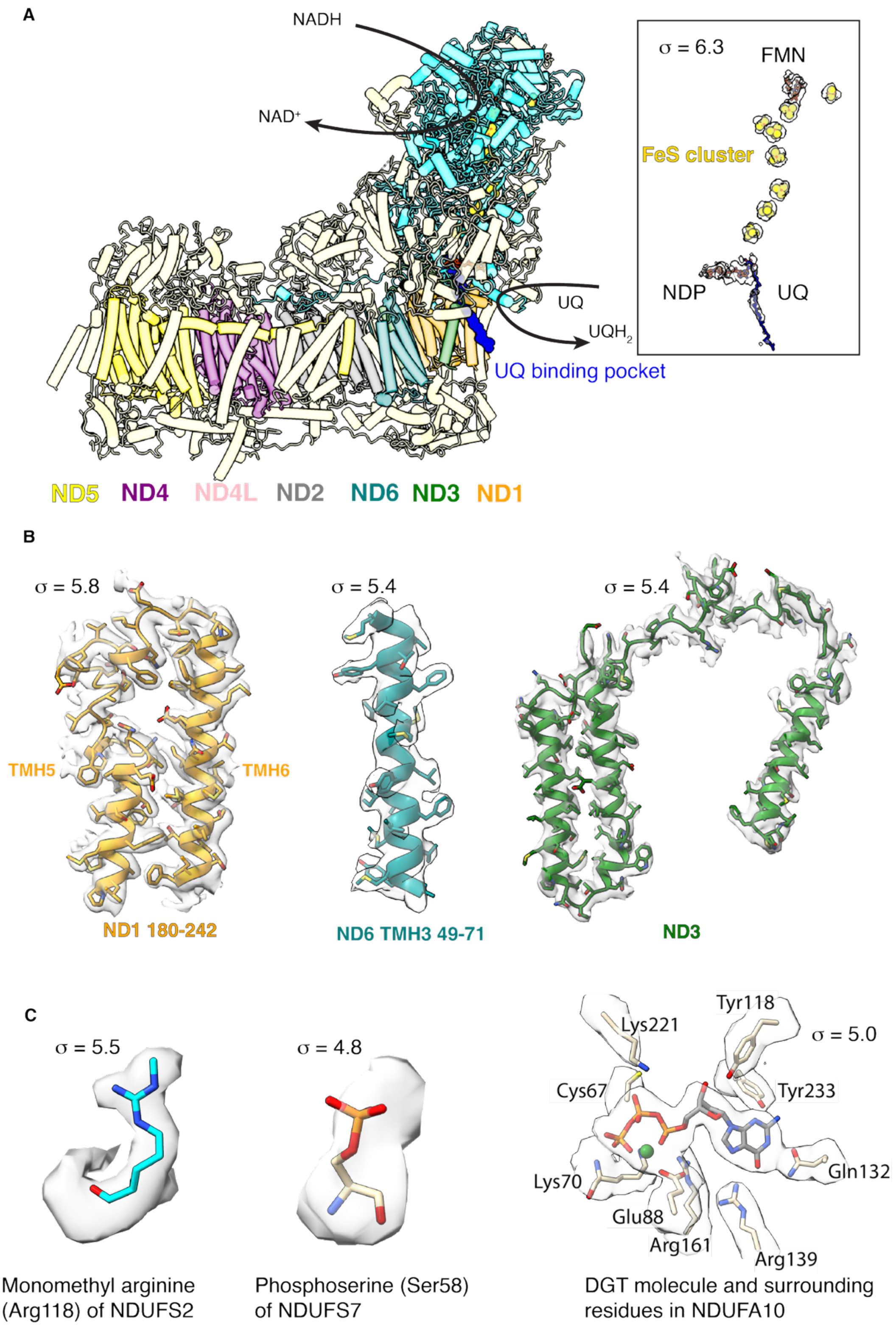

Supplementary Figure 3. Overall structure of complex I and map quality of its key features

(A) Overall structure of human complex I in this study. All subunits are displayed in cartoon representation with the 7 hydrophilic core subunits in cyan and the 31 supernumerary subunits in wheat. The seven transmembrane core subunits (mtDNA encoded proteins) are colored as labeled (ND5: yellow; ND4, purple; ND2, gray; ND4L,

pink; ND6, teal; ND3, green; ND1, orange). Electrons are transferred from NADPH (NDP) to the flavin mononucleotide (FMN) in the hydrophilic domain and then sequentially down a series of Fe-S clusters (orange-yellow spheres). The terminal Fe-S cluster N2, situated above the membrane, reduces the membrane-bound ubiquinone-10 (UQ) substrate to ubiquinol (UQH2) in a binding channel (blue surface) at the interface of the hydrophilic and membrane domains. The inset shows the electron density for FMN, Fe-S clusters, and NDP molecules. Interestingly, a broken density is observed at the ubiquinone (blue stick) binding site, suggesting a mixed occupancy in our map. The contour levels of the maps (σ) are indicated in the panel.

(B) The cryo-EM densities of the auto-sharpened map display the ordered nature of the ND1-TMH5-6 and ND3-TMH1-2 loops and the a-helical structure of ND6-TMH3, which are all specific to the active state. The contour levels of the maps (σ) are indicated in the panel.

(C) The densities of post-translational modifications of monomethylated NDUFS2-Arg118, the phosphoserine of Ser58 of NDUFS7, and the dGTP-Mg^{2+} bound in NDUFA10 with surrounding residues. The contour levels of the maps (σ) are indicated in the panel.

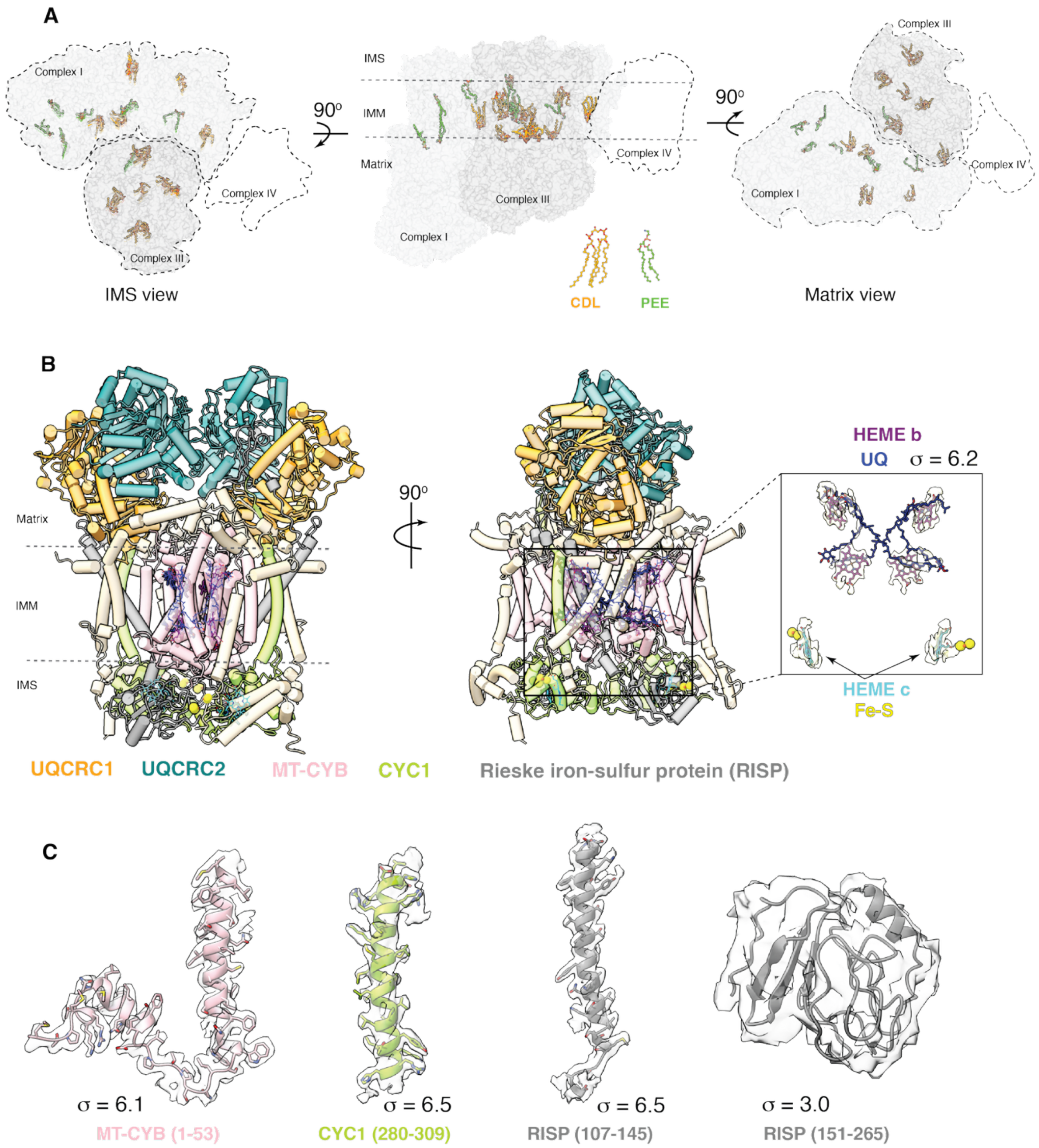

Supplementary Figure 4. Overall structure of complex III and map quality of its key features

(A) The distribution of modeled common lipid molecules, including cardiolipin (coral) and PEE (lime) in CI and CIII. The CI and $CIII_2$ are shown as surface and the relative position of CIV is indicated.

(B) Overall structure of CIII dimer. All subunits are displayed in cartoon representation, with the five supernumerary subunits in wheat. The IMM, matrix and IMS are indicated. The five core subunits are colored as labeled (UQCRC1: orange; UQCRC2: teal; MT-CYB, a mt-DNA encoded protein: pink; CYC1: green; Rieske iron-sulfur protein (RISP): grey). Heme b_L and b_H, UQ, and Heme c_1 are shown as purple, blue and cyan sticks, respectively. The Fe-S clusters are shown as orange-yellow spheres. The inset shows

the electron densities for four Heme and UQ molecules. The contour levels of the maps (σ) are indicated in the panel.

(C) The cryo-EM densities from the auto-sharpened map for several representative domains. The well-resolved densities were observed for MT-CYB (1-53), representative membranous helices of CYC1 (280-309) and RISP (107-145), while a poorly resolved map was observed for the IMS-facing domain of RISP (151-265). The contour levels of the maps (σ) are indicated in the panel.

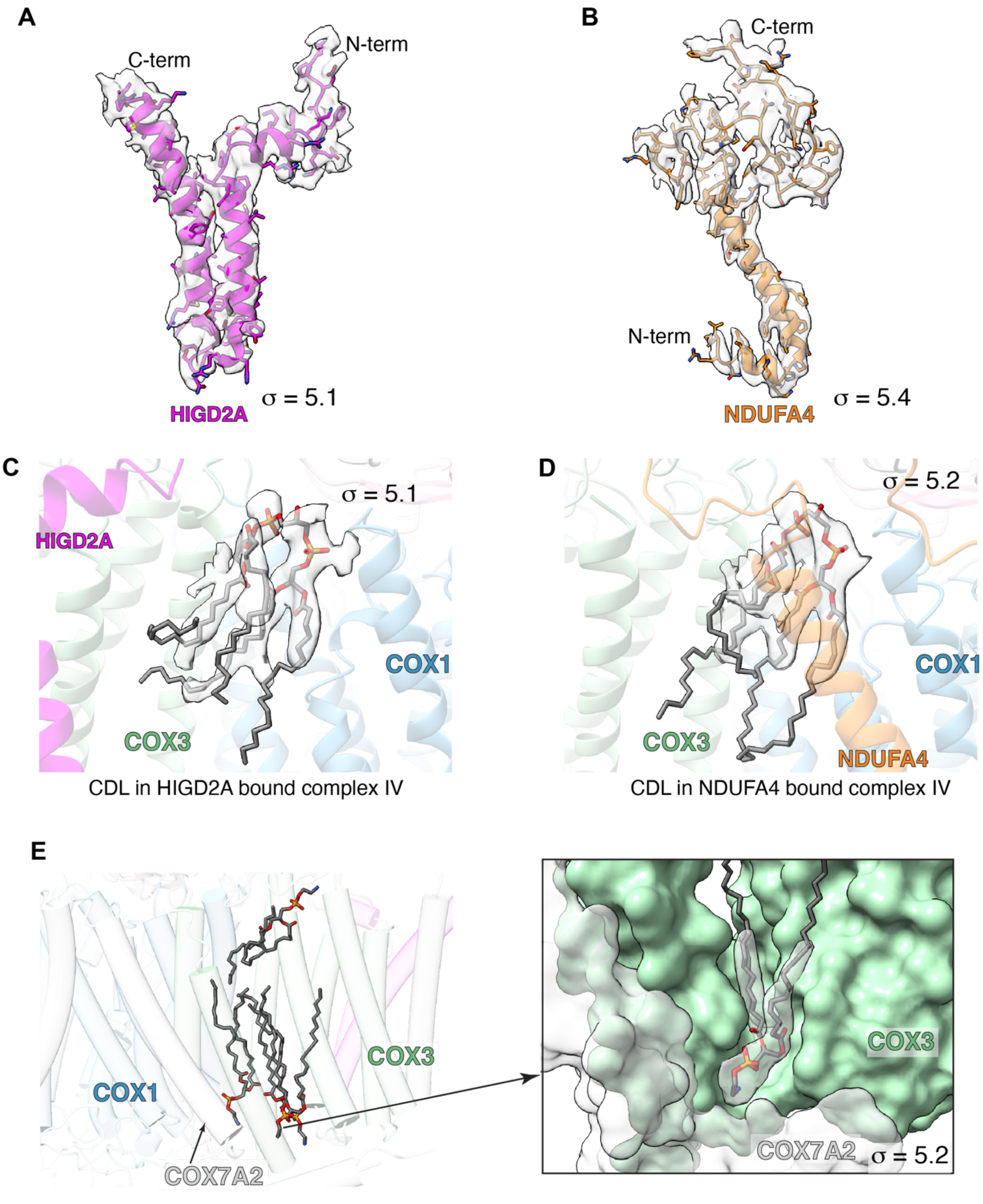

Supplementary Figure 5. Cryo-EM densities of key features of complex IV in this study

The densities of **(A)** HIGD2A, **(B)** NDUFA4, and cardiolipin molecules in **(C)** HIGD2A-bound and **(D)** NDUFA4-bound complex IV. The contour levels of the maps (σ) are indicated in the panel.

(E) Position of four phosphatidylethanolamine (PEE) molecules within COX3. The interface between COX1 and COX3 is shown (left panel). Three of these PEE

molecules have been previously observed [1]; the density for the fourth one, which is located between COX3 and COX7A2 (shown as surface), is shown in the right panel.

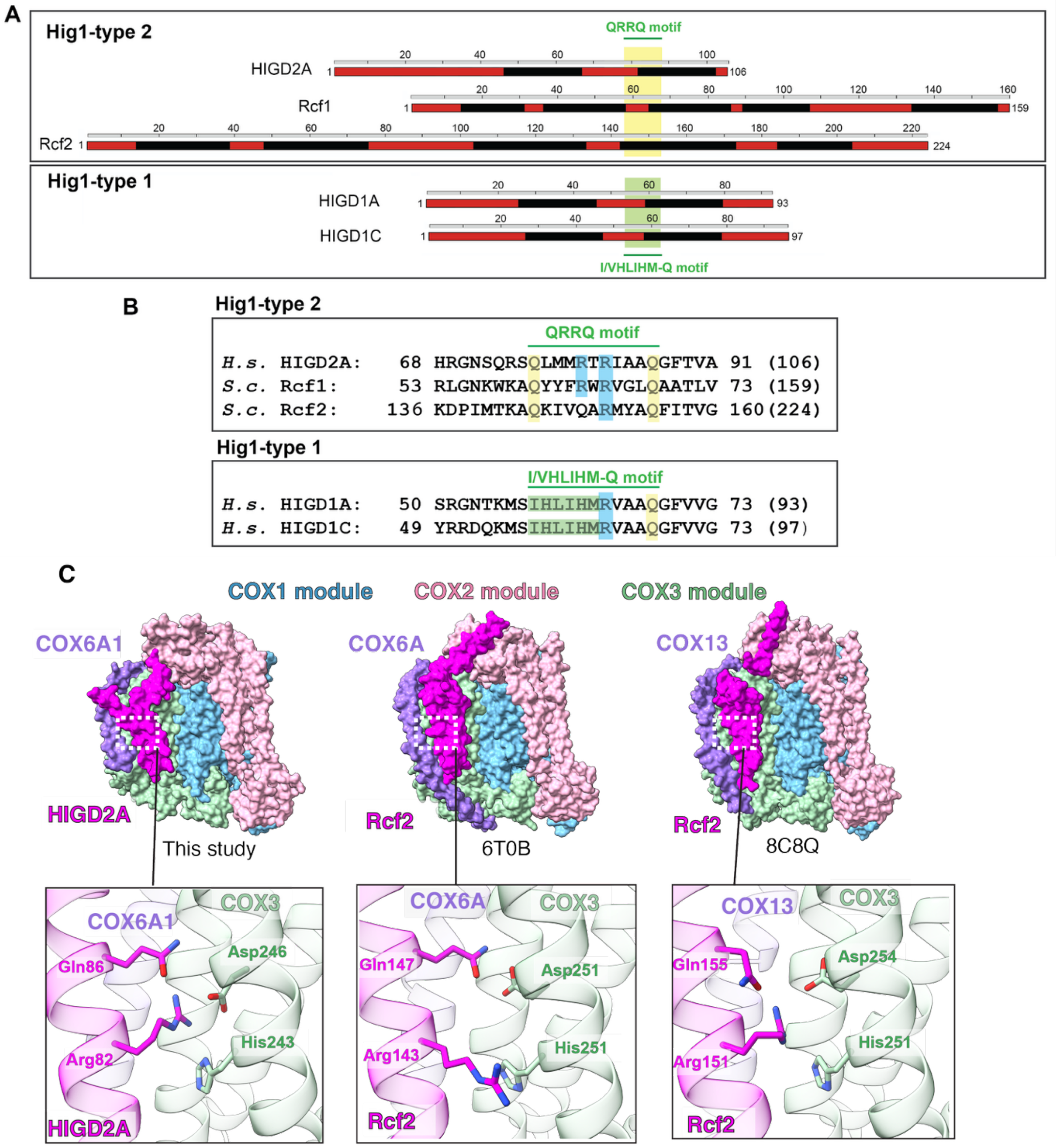

Supplementary Figure 6. Structural comparison and sequence analysis of Hig1 domain protein family from human and yeast

(**A**) Alignment of the human and yeast Hig1 domain protein family members according to the predicted position of their functional QRRQ (in type 2 proteins) or I/VHLIHM-Q (in type 1 proteins) motifs. Transmembrane domains are indicated in black. The schematic representation of the domains was retrieved from the UniProt database for the proteins HIGD2A (Ref. Q9BW72), Rcf1 (Ref. Q03713), Rcf2(Ref. P53721), HIGD1A (Ref. Q9Y241), and HIGD1C (Ref. A8MV81).

(**B**) Sequence alignment of the Hig1 domain protein conserved motifs QRRQ (type 2) and I/VHLIHM-Q (type 1) in human and yeast proteins.

(C) Upper panel: the structures of HIGD2A bound human CIV within the respirasome (this study, left panel), Rcf2 bound CIII2-CIV2 (the second CIV and $CIII_2$ are hidden for

better visualization) (PDB: 6T0B[2], middle panel) from *Saccharomyces cerevisiae* (*S.cerevisiae*) and Rcf2 bound CIV (PDB: 8C8Q[3], right panel) from *Schizosaccharomyces pombe (S.pombe)*. Lower panel: the close view of the interactions between the third Arg and fourth Gln residues from the conserved motif (QRRQ) in Hig1 domain proteins (type 2) and the conserved Asp and His residues from COX3 protein. This view is same as shown in **Figure 2B**. Lower panel: The HIGD2A and Rcf2 proteins are shown as magenta; COX3 proteins are shown as green; COX6A1 (from HIGD2A bound CIV, this study), COX6A (in SC: $CIII_2CIV_2$ from *S.cerevisia*) and COX13 (in CIV from *S.pombe*) are shown as violet. Upper panel: Proteins which belong to COX1, COX2 and COX3 modules are colored as cyan, pink and light green, respectively.

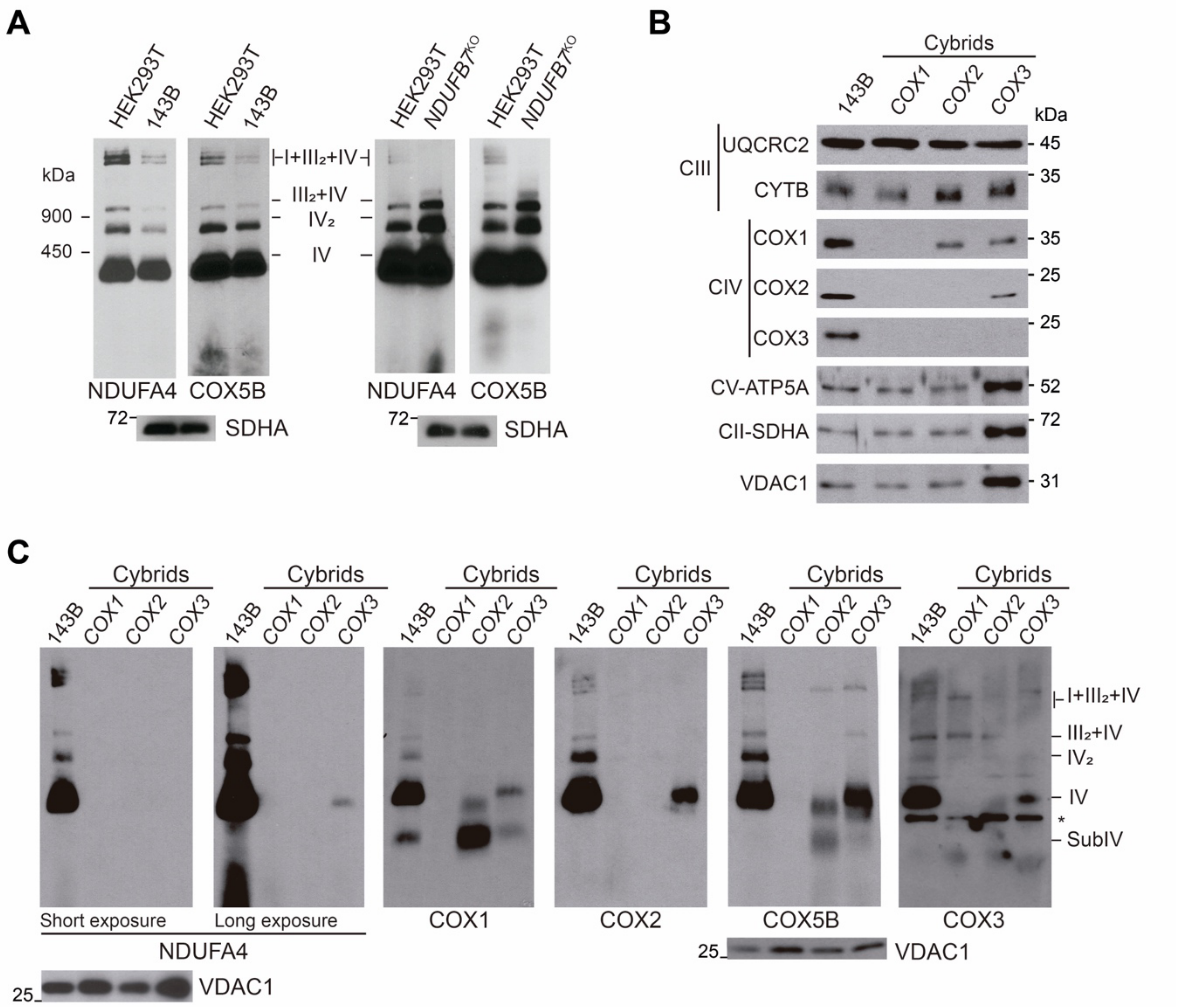

Supplementary Figure 7. NDUFA4 is a late assembly CIV subunit

(**A** and **C**) BN-PAGE coupled with immunoblotting analyses of NDUFA4 and MRC CIV markers distribution in isolated complex, supercomplexes, and assembly intermediates in (**A**) HEK293T cells wild-type or KO for CI subunit *NDUFB7* and 143B cells or (**C**) 143B cells wild-type or cybrid lines carrying homoplasmic mutations in *COX1, COX2*, or *COX3*. Purified mitochondria were extracted with digitonin (detergent:protein ratio 4 g/g). Antibodies against SDHA or VDAC were used as a loading control. *COX3 antibody unspecific signal.

(**B**) Steady-state levels of the indicated MRC complex subunits assessed by SDS-PAGE followed by immunoblotting in mitochondria purified from 143B cells and *COX1, COX2,* or *COX3* mutant cybrids. All the experiments in this figure were repeated three times with similar results. Source data are provided as a Source Data file.

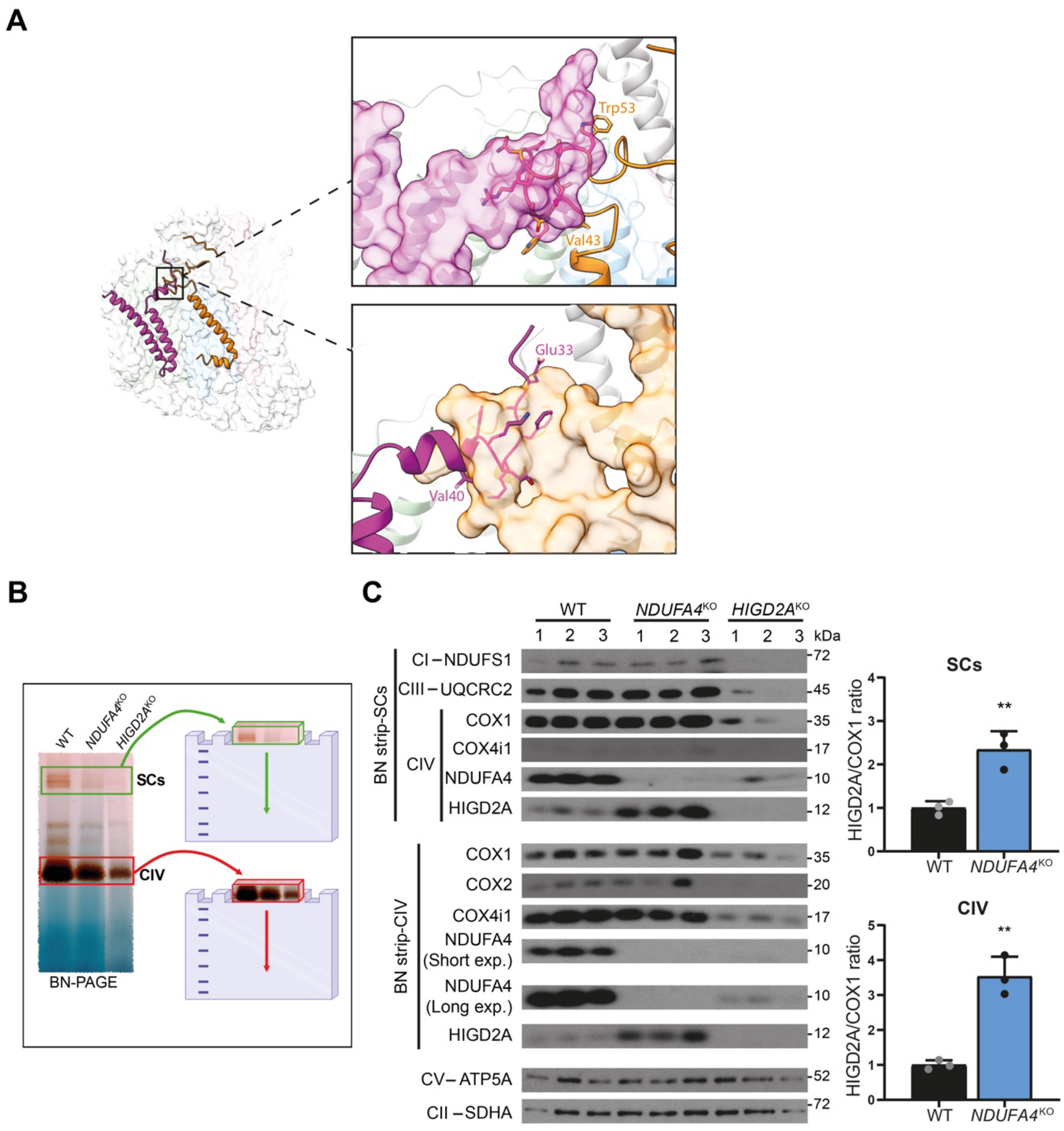

Supplementary Figure 8. Stable binding of HIGD2A to isolated and supercomplexed CIV is increased in *NDUFA4*^{KO} cells

(A) Structural details of the clashing regions between HIGD2A and NDUFA4. The superimposition of two structures of class 1 and 4 in this study shows the residues, which are shown as sticks, of NDUFA4 (upper panel, orange, from Val43 to Trp53) and HIGD2A (lower panel, magenta, from Glu33 to Val40) involved in the clashing regions between the two proteins.

(**B**) Experimental outline of a two-dimensional (2D) BN-PAGE / SDS-PAGE analysis of isolated and supercomplexed CIV composition. Created with BioRender. Sierra, A. (2025) https://BioRender.com/0ey1ze6.

(**C**) Following BN-PAGE of digitonin solubilized mitochondria (digitonin: protein ratio of 4 g/g) isolated from HEK wild type (WT), *NDUFA4*^{KO,} and *HIGD2A*^{KO} cells, the bands corresponding to CIV or supercomplexes (SCs) I+III$_2$+IV$_n$ were excised from the gel and run on a second dimension SDS-PAGE. The HIGD2A signal was normalized by

the COX1 signal and plotted in the bar graph relative to WT (mean ± SD of 3 independent experiments). Dots represent individual data points. T-test (two-sided). **$p<0.01$. Upper graph $p=0.0066$, lower graph $p=0.0016$. Exact test statistics are provided in Source Data Table.

A

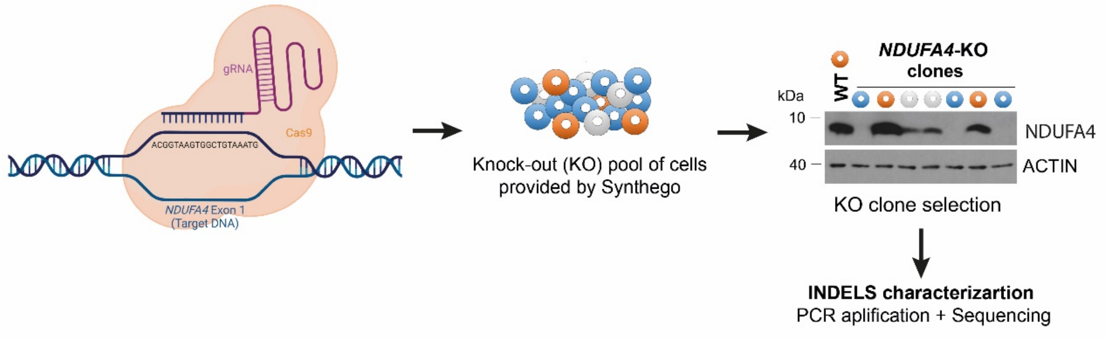

B

CRISPR-Cas9 edited gene	Genotype	DNA	Protein
NDUFA4	C. Heterozygous	c.1A>G, c.4insT	No start codon

Supplementary Figure 9. Workflow followed for the generation, identification and characterization of *NDUFA4*^{KO} clones
(A) The CRISPR-Cas9 gene editing system employed by Synthego to induce mutations in Exon 1 of the *NDUFA4* gene in HEK293T cells. A pool of cells containing *NDUFA4* knockouts (KO), was subjected to clonal selection. NDUFA4-depleted clones were selected and characterized by studying insertions or deletions (INDELS) in the *NDUFA4* gene through PCR amplification of the *NDUFA4* locus, followed by sequencing. Created in BioRender. Sierra, A. (2025) https://BioRender.com/6h678z2.
(B) Table detailing the genotype of the HEK293T cell line carrying edited *NDUFA4* used for this study. C. Hetero, compound heterozygous.

TABLES

Supplementary Table 1: Cryo-EM data collection, refinement and validation statistics

Data collection, processing and refinement	Complex I	Complex III	HIGD2A-bound complex IV (class 1)	NDUFA4-bound complex IV (class 4)
PDB/EMDB	9TI4/ EMDB-54784	9HZL/EMD-52525	9I6F/EMD-52654	9I7U/EMD-52662
**Data collection and processing**				
Microscope	Titan Krios G3i			
Detector	Gatan K3			
Magnification	105,000			
Voltage (kV)	300			
Total electrons expose (e^-/Å^2)	35			
Defocus range (μM)	-0.6 to -2.0			
Symmetry imposed	C1			
Processed particles (no.)	301,780			
Final particles	236,246	213,731	131,793	109,624
Map resolution (Å) (local refinement)	2.66	2.52	2.93	3.15
FSC threshold	0.143	0.143	0.143	0.143
Map-sharpening B-factor (Å^2)	-83.3	-76.3	-89.90	-68.67
**Refinement**				
Initial model used (PDB)	5XTD	5XTE	5Z62	5Z62
Model composition				
Total atom (hydrogen)	136483 (68786)	68734 (34118)	30359 (15213)	15296 (0)
Chains	45	10	14	14
Protein residues	8244	4209	1835	1848
Metal ion	Mg^{2+}:1<br>Zn^{2+}:1		Mg^{2+}:1<br>Zn^{2+}:1<br>Cu^{2+}: 3	Mg^{2+}:1<br>Zn^{2+}:1<br>Cu^{2+}: 3
Ligands	CDL:6; SF4:6; PLX:7; FMN:1; FES:2; DGT:1; PEE:7; 8Q1:2; NDP:1;	CDL:9; PC1:1; PLX:2; FES:2; HEC:2; U10:4; PEE:1; 3PE:6; HEM:4	HEA:2; CDL:1; PEE:4; PGV:1	HEA:2; CDL:1; PEE:4; PGV:1
Model to map CC (CCmask/CCbox/CCpeaks/CCvolume)	0.83/0.67/0.52/0.83	0.82/0.66/0.54/0.81	0.78/0.63/0.55/0.76	0.79/0.67/0.60/0.77
Resolution (Å) by model-to-map FSC, threshold 0.50 (masked/Unmasked)	2.8/3.3	2.8/3.1	3.0/3.2	3.2/3.3
Average B factors (Å^2)				
Protein	92.06	74.18	99.74	93.87
Ligand	104.24	76.90	91.79	84.81
R.M.S. deviations				
Bond lengths (Å)	0.004	0.003	0.003	0.003
Bond angles (°)	0.688	1.163	0.819	0.950
**Validation**				
Clash score	4.9	6.81	7.97	6.04
Poor Rotamers (%)	0.00	0.03	0.00	0.00
Ramachandran Plot				
Outliers (%)	0.14	0.00	0.06	0.30
Allowed (%)	6.60	3.75	6.64	5.72
Favored (%)	93.26	96.25	93.30	93.98
MolProbility score	1.69	1.63	1.87	1.74

Supplementary Table 2: Summary of model building for human complex I

Subunit	Alternative names	Chain	Total residues*	Modelled residues (%)	Modelled cofactor and modification
NDUFV1	51 kDa, Nqo1, NuoF	C	21-464	27-460 (97.7)	FMN, 4Fe4S
NDUFV2	24 kDa, Nqo2, NuoE	O	33-249	36-247 (97.7)	2Fe2S
NDUFV3	10 kDa	K	35-108	68-106 (52.7)	
NDUFS1	75 kDa, Nqo3, NuoG	M	24-727	30-716 (94.9)	2Fe2S, 2 x 4Fe4S
NDUFS2	49 kDa, Nqo4, NuoC	Q	34-463	34-463 (100)	Monomethyl-Arg118
NDUFS3	30 kDa, Nqo5, NuoC	P	37-264	43-250 (91.2)	
NDUFS4	18 kDa	L	43-175	55-175 (90.3)	
NDUFS5	15 kDa	h	1-106	1-105 (99)	C33-C66<br>C43-C56
NDUFS6	13 kDa	T	29-124	29-124 (100)	Zn^{2+}
NDUFS7	PSST, Nqo6, NuoB	E	39-213	53-213 (83.4)	4Fe4S, Phospho-Ser58
NDUFS8	TYKY, Nqo9, NuoI	D	35-210	35-210 (100)	2 x 4Fe4S
ND1	Nqo8, NuoH	s	1-318	3-318 (99.4)	
ND2	Nqo14, NuoN	i	1-347	1-347 (100)	
ND3	Nqo7, NuoA	j	1-115	1-115 (100)	
ND4	Nqo13, NuoM	r	1-459	1-459 (100)	
ND4L	Nqo11, NuoK	k	1-98	1-98 (100)	
ND5	Nqo12, NuoL	l	1-603	3-603 (99.7)	
ND6	Nqo10, NuoJ	m	1-174	1-174 (100)	
NDUFA1	MWFE	S	1-70	1-70 (100)	
NDUFA2	B8	G	2-99	14-98 (86.7)	
NDUFA3	B9	U	2-84	2-84 (100)	
NDUFA5	B13	I	2-116	5-116 (97.4)	
NDUFA6	B14	F	1-128	11-128 (92.2)	
NDUFA7	B14.5a	t	2-113	2-74; 90-113 (86.6)	
NDUFA8	PGIV	u	2-172	2-172 (100)	C36-C66
NDUFA9	39 kDa	J	36-377	36-376 (99.7)	NDP
NDUFA10	42 kDa	w	36-355	36-355 (100)	Mg^{2+}-dGTP
NDUFA11	B14.7	V	2-141	2-141 (100)	C95-C105
NDUFA12	B17.2	N	1-145	1-144 (99.3)	
NDUFA13	B16.6	W	2-144	30-144 (80.4)	
NDUFAB1a	Acyl carrier protein, SDAPa	H	69-156	72-156 (96.6)	S-dodecanoyl-4'-phosphopantetheine
NDUFAB1b	Acyl carrier protein, SDAPb	X	69-156	71-156 (97.7)	S-dodecanoyl-4'-phosphopantetheine
NDUFB1	MNLL	n	2-58	3-58 (98.2)	
NDUFB2	AGGG	Y	34-105	37-97 (84.7)	
NDUFB3	B12	Z	2-98	10-92 (84.5)	
NDUFB4	B15	o	2-129	2-129 (100)	
NDUFB5	SGDH	a	47-189	52-189 (96.5)	
NDUFB6	B17	b	2-128	2-124 (96.9)	
NDUFB7	B18	v	2-137	2-124 (90.4)	C69-C80
NDUFB8	ASHI	c	29-186	31-182 (96.2)	
NDUFB9	B22	p	2-179	7-179 (97.2)	
NDUFB10	PDSW	d	1-172	3-170 (97.7)	C71-C78
NDUFB11	ESSS	e	30-153	44-151 (87.1)	
NDUFC1	KFYI	f	27-76	27-75 (98)	
NDUFC2	B14.5b	g	1-119	1-119 (100)	

*Total residues: the sequence from Uniprot without the mitochondrial translational sequence (MTS)

Supplementary Table 3: Summary of model building for human complex III in this study

Subunit	Alternative names	Chain	Total residues*	Modelled residues	Modelled cofactor and modification
Cytochrome b-c1 complex subunit 1, mitochondrial	Complex III subunit 1, UQCRC1	L, Y	1-480	35-480	
Cytochrome b-c1 complex subunit 2, mitochondrial	Complex III subunit 2, UQCRC2	K, W	1-453	35-453	
Cytochrome b	Complex III subunit 3, MT_CYB	J, V	1-380	1-379	4 heme, 4UQ binding
Cytochrome c1	Complex III subunit 4, CYC1	H, U	85-325	85-325	2HEC
Cytochrome b-c1 complex subunit Rieske	Complex III subunit 5; Rieske iron-sulfur protein (RISP) UQCRFS1	B, C, O, P	1-274	1-57<br>79-274	2x 2Fe-2S
Cytochrome b-c1 complex subunit 7, mitochondrial	Complex III subunit 7, UQCCRB	F, S	2-111	6-111	
Cytochrome b-c1 complex subunit 8, mitochondrial	Complex III subunit 8, UQCRQ	A, N	2-82	2-82	
Cytochrome b-c1 complex subunit 6, mitochondrial	Complex III subunit 6, UQCRH	E, R	14-91	19-91	
Cytochrome b-c1 complex subunit 9	Ubiquinol-cytochrome c reductase complex 7.2 kDa protein, UQCR10	D, Q	2-63	2-63	
Cytochrome b-c1 complex subunit 10	Ubiquinol-cytochrome c reductase complex 6.4 kDa protein, UQCR11	G, T	1-56	2-52	

*Total residues: the sequence from Uniprot without the mitochondrial translational sequence (MTS)

Supplementary Table 4: Summary of model building for human complex IV in this study

Subunit	Gene name	Chain	Total residues*	Modelled residues	Modelled cofactor and modification	Note
Cytochrome c oxidase subunit 1	MT-CO1	A	1-513	1-513	2 Heme A, Cu^{2+},Mg^{2+}, fMet	
Cytochrome c oxidase subunit 2	MT-CO2	B	1-453	35-453		
Cytochrome c oxidase subunit 3	MT-CO3	C	1-380	1-379	fMet	
Cytochrome c oxidase subunit 4 isoform 1	COX4i1	D	23-169	31-169		
Cytochrome c oxidase subunit 5A	COX5A	E	42-150	46-150		
Cytochrome c oxidase subunit 5B	COX5B	F	32-129	34-129	Zn^{2+}	
Cytochrome c oxidase subunit 6A1	COX6A1	G	25-109	34-108		
Cytochrome c oxidase subunit 6B1	COX6B1	H	2-86	5-86	C30-C65<br>C40-C54	
Cytochrome c oxidase subunit 6C	COX6C	I	1-75	4-75		
Cytochrome c oxidase subunit 7A2	COX7A2	J	24-83	24-80		
Cytochrome c oxidase subunit 7B	COX7B	K	25-80	27-79		
Cytochrome c oxidase subunit 7C	COX7C	L	17-63	17-63		
Cytochrome c oxidase subunit 8A	COX8A	M	26-69	26-68		
HIG1 domain family member 2A	HIGD2A	N	2-106	31-106		HIGD2A-complex IV (class 1)
Cytochrome c oxidase subunit NDUFA4	NDUFA4	N	1-81	1-81		NDUFA4-complex IV (class 4)

*Total residues: the sequence from Uniprot without the mitochondrial translational sequence (MTS)

Supplementary Table 5. Oligonucleotides

Name	Sequence
SeqNDUFA4-F	GGCCACCAGACTGTTCCAAT
SeqNDUFA4-R	GAGGTCCTGGGTGACTTTGG
InfusionpHygro-AsisI-HIGD2AdCR-F	AGATCTGCCGCCGCGATCGCCGCAAGACCCGCGAGAAC
InfusionpHygro-PmeI-HIGD2AdCR-R	CGCGGCCGGCCGTTTAAACCTTATCGTCGTCATCCTTGTAATCC
HIGD2A-MutCR_F	AGCGCAAGCTTCGTTCGCAAGACCCGC
HIGD2A-MutCR_R	GAAACTCGCTGGATTCCTGTAAACAGTGGG
NDUFA4-dCR-F	TAAATGAAATGTTTCACTATAACG
NDUFA4-dCR-R	ATCTGGATTGAACAATGC
NDUFA4-MutCR_F	AGCAATAACCCAGCCCACTGGAACAAACTGGGTCCC
NDUFA4-MutCR_R	GTCGGAACAAACATCATGATTGAACAATGCCAGACG
COX14-F	ATTGGATCCATGCCAACTGGCAAG
COX14-R	AATCTCGAGCTACTTATCGTCGTCATCCTTGTAATCCATG

Supplementary Table 6. Plasmids

Plasmid name	Source	Catalog number
HIGD2A- Myc-DDK in pCMV6-Entry	Origene	RC201223
NDUFA4 in pCMV6-XL5	Origene	SC118617
COX14::FLAG in pcDNA5/FRT/TO	In house	In house
pCMV6-A-Entry-Hygro	Origene	PS100024

Supplementary Table 7. List of antibodies

Target	Source	Identifier
ATP5A	Abcam	Cat# ab14748, RRID: AB_301447
SDHA	Proteintech	Cat#14865-1-AP, RRID:AB_11182164
NDUFS1	Thermo Fisher Scientific	Cat# PA5-22309, RRID: AB_11151879
NDUFA9	Abcam	Cat# ab14713, RRID: AB_301431
NDUFB11	Abcam	Cat# ab183716, RRID: AB_2298378
MTCYB	Proteintech	Cat# 55090-1-AP, RRID: AB_2881266
UQCRFS1 (RISP)	Abcam	Cat# ab14746, RRID: AB_301445

Target	Source	Identifier
UQCRC2	Abcam	Cat# ab14745, RRID: AB_2213640
COX1	Abcam	Cat# ab14705, RRID: AB_2084810
COX2	Abcam	Cat# ab110258, RRID: AB_10887758
COX3	Abcam	Cat# ab110259, RRID: AB_10859925
COX4I1	Abcam	Cat# ab14744, RRID: AB_301443
COX5B	Santa Cruz	Cat# sc-374417, RRID: AB_10988066
COX6A1	Sigma	Cat# HPA062394, RRID: AB_2684749
NDUFA4	Origene	Cat# TA351429
COX14	Provided by Peter Rehling	Ritcher-Dennerlein et al, 2016 [4] Haga clic o pulse aquí para escribir texto.
HIGD2A	Sigma	Cat# HPA042715, RRID: AB_2678127
VDAC1	Abcam	Cat# ab14734, RRID: AB_443084
TOM20	SantaCruz	Cat# sc-11415, RRID: AB_2207533
TIMM17	Abcam	Cat# ab192246
2° Ab-mouse	Rockland Immunochemicals	Cat# 610-103-121 RRID: AB_218457
2° Ab-rabbit	Rockland Immunochemicals	Cat# 611-1302 RRID: AB_219720

Supplementary References

1 Zong, S. *et al*. Structure of the intact 14-subunit human cytochrome c oxidase. *Cell Res*. **28**, 1026-1034, doi:10.1038/s41422-018-0071-1 (2018).

2 Hartley, A. M., Meunier, B., Pinotsis, N. & Maréchal, A. Rcf2 revealed in cryo-EM structures of hypoxic isoforms of mature mitochondrial III-IV supercomplexes. *Proc. Natl. Acad. Sci. USA* **117**, 9329-9337, doi:doi:10.1073/pnas.1920612117 (2020).

3 Moe, A., Ädelroth, P., Brzezinski, P. & Näsvik Öjemyr, L. Cryo-EM structure and function of S. pombe complex IV with bound respiratory supercomplex factor. *Communications Chemistry* **6**, 32, doi:10.1038/s42004-023-00827-3 (2023).

4 Richter-Dennerlein, R. *et al*. Mitochondrial Protein Synthesis Adapts to Influx of Nuclear-Encoded Protein. *Cell* **167**, 471-483.e410, doi:10.1016/j.cell.2016.09.003 (2016).
