# Supplementary material for: Structural basis for late maturation steps of mitochondrial respiratory chain complex IV within the human respirasome
Source: Nat Commun. 2026 Jan 10;17:1550. doi: 10.1038/s41467-025-68274-3 (PMC12894743; doi:10.1038/s41467-025-68274-3)
Supplement: Supplementary file 2 — Description of Additional Supplementary Files [file 41467_2025_68274_MOESM2_ESM.pdf]

Description of Additional Supplementary Files

File name: Supplementary Movie 1
Description: 3D variability analysis showing the conformational variation of complex IV relative to complex I and complex III (colors as in Figure 1). The mask covering complex I is applied for 3D variability analysis. The output mode was set as 'simple', number of frames were set as 20.
