# Supplementary material for: Structural basis for late maturation steps of mitochondrial respiratory chain complex IV within the human respirasome
Source: Nat Commun. 2026 Jan 10;17:1550. doi: 10.1038/s41467-025-68274-3 (PMC12894743; doi:10.1038/s41467-025-68274-3)
Supplement: Supplementary file 4 — Reporting Summary [file 41467_2025_68274_MOESM4_ESM.pdf]

nature portfolio

Corresponding author(s): Joanna Rorbach
Last updated by author(s): 2025.11.19

Reporting Summary

Nature Portfolio wishes to improve the reproducibility of the work that we publish. This form provides structure for consistency and transparency in reporting. For further information on Nature Portfolio policies, see our Editorial Policies and the Editorial Policy Checklist.

Statistics

For all statistical analyses, confirm that the following items are present in the figure legend, table legend, main text, or Methods section.

n/a	Confirmed	
☐	☒	The exact sample size (*n*) for each experimental group/condition, given as a discrete number and unit of measurement
☐	☒	A statement on whether measurements were taken from distinct samples or whether the same sample was measured repeatedly
☐	☒	The statistical test(s) used AND whether they are one- or two-sided<br>*Only common tests should be described solely by name; describe more complex techniques in the Methods section.*
☒	☐	A description of all covariates tested
☒	☐	A description of any assumptions or corrections, such as tests of normality and adjustment for multiple comparisons
☐	☒	A full description of the statistical parameters including central tendency (e.g. means) or other basic estimates (e.g. regression coefficient) AND variation (e.g. standard deviation) or associated estimates of uncertainty (e.g. confidence intervals)
☐	☒	For null hypothesis testing, the test statistic (e.g. *F*, *t*, *r*) with confidence intervals, effect sizes, degrees of freedom and *P* value noted<br>*Give P values as exact values whenever suitable.*
☒	☐	For Bayesian analysis, information on the choice of priors and Markov chain Monte Carlo settings
☒	☐	For hierarchical and complex designs, identification of the appropriate level for tests and full reporting of outcomes
☒	☐	Estimates of effect sizes (e.g. Cohen's *d*, Pearson's *r*), indicating how they were calculated

Our web collection on statistics for biologists contains articles on many of the points above.

Software and code

Policy information about availability of computer code

Data collection	As described in the methods: Cryo-EM data were collected from vitrified grids using a Krios G3i electron microscope (Thermofisher) operated at 300 kV and equipped with a K3 Bioquantum detector (Gatan). Automated data collection software (EPU, Thermofisher) was used during collection which was performed at 105,000x EFTEM SA magnification, yielding a calibrated pixel size of 0.84 Å.
Data analysis	CryoEM data were processed and analyzed using cryoSPARC v3.2-v4.2, COOT 0.8.9, Phenix 1.21rc-1, Chimera 1.16, as described in methods.

For manuscripts utilizing custom algorithms or software that are central to the research but not yet described in published literature, software must be made available to editors and reviewers. We strongly encourage code deposition in a community repository (e.g. GitHub). See the Nature Portfolio guidelines for submitting code & software for further information.

Data

Policy information about availability of data

All manuscripts must include a data availability statement. This statement should provide the following information, where applicable:

- Accession codes, unique identifiers, or web links for publicly available datasets
- A description of any restrictions on data availability
- For clinical datasets or third party data, please ensure that the statement adheres to our policy

All cryo-EM maps and atomic coordinates have been deposited at the Electron Microscopy Data Bank (EMDB) and the Protein Data Bank (PDB) as follows: EMD-52596 (initial consensus map); complex I: EMD-52619/PDB 9I4I; complex III EMD-52525/PDB 9HZL; complex IV: EMD-52664 (initial local refinement of

consensus map), different states of complex IV: EMD-52654/PDB 9I6F (class 1: HIGD2A bound complex IV), EMD-52612 (class 2: complex IV with HIGD2A density and incoming NDUFA4), EMD-52613 (class 3: NDUFA4 bound complex IV with weak density from HIGD2A), EMD-52662/PDB 9I7U (class 4: NDUFA bound complex IV).

Research involving human participants, their data, or biological material

Policy information about studies with human participants or human data. See also policy information about sex, gender (identity/presentation), and sexual orientation and race, ethnicity and racism.

Reporting on sex and gender	N/A
Reporting on race, ethnicity, or other socially relevant groupings	N/A
Population characteristics	N/A
Recruitment	N/A
Ethics oversight	N/A

Note that full information on the approval of the study protocol must also be provided in the manuscript.

Field-specific reporting

Please select the one below that is the best fit for your research. If you are not sure, read the appropriate sections before making your selection.

☒ Life sciences ☐ Behavioural & social sciences ☐ Ecological, evolutionary & environmental sciences

For a reference copy of the document with all sections, see nature.com/documents/nr-reporting-summary-flat.pdf

Life sciences study design

All studies must disclose on these points even when the disclosure is negative.

Sample size	Numbers of particles in the cryoEM samples are described in the methods
Data exclusions	CryoEM data processing, including which particles are included or excluded from the final 3D map, is described in the methods.
Replication	Two cryoEM data sets were collected from a single grid. As a result, cryoEM structure determinations were not repeated, as is standard in the field due to the large amount of time and effort involved in each structure determination. Western blotting and Blue native experimnets were performed at least 3 times. All attempts in replication were successful.
Randomization	CryoEM particles were randomly assigned to two half-sets, following the "gold standard" FCS protocol.
Blinding	Blinding is not relevant to the cryoEM technique used in this study; none of the information collected and processed is subjected to experimenter bias in a way that blinding would address.

Reporting for specific materials, systems and methods

We require information from authors about some types of materials, experimental systems and methods used in many studies. Here, indicate whether each material, system or method listed is relevant to your study. If you are not sure if a list item applies to your research, read the appropriate section before selecting a response.

Materials & experimental systems

n/a	Involved in the study	
☐	☒	Antibodies
☐	☒	Eukaryotic cell lines
☒	☐	Palaeontology and archaeology
☒	☐	Animals and other organisms
☒	☐	Clinical data
☒	☐	Dual use research of concern
☒	☐	Plants

Methods

n/a	Involved in the study	
☒	☐	ChIP-seq
☒	☐	Flow cytometry
☒	☐	MRI-based neuroimaging

Antibodies

Antibodies used	All antibodies used in this study are listed in the Table S7<br>ATP5A Abcam Cat# ab14748, RRID: AB_301447<br>SDHA Proteintech Cat#14865-1-AP, RRID:AB_11182164<br>NDUFS1 Thermo Fisher Scientific Cat# PA5-22309, RRID: AB_11151879<br>NDUFA9 Abcam Cat# ab14713, RRID: AB_301431<br>NDUFB11 Abcam Cat# ab183716, RRID: AB_2298378<br>MTCYB Proteintech Cat# 55090-1-AP, RRID: AB_2881266<br>UQCRFS1 (RISP) Abcam Cat# ab14746, RRID: AB_301445<br>UQCRC2 Abcam Cat# ab14745, RRID: AB_2213640<br>COX1 Abcam Cat# ab14705, RRID: AB_2084810<br>COX2 Abcam Cat# ab110258, RRID: AB_10887758<br>COX3 Abcam Cat# ab110259, RRID: AB_10859925<br>COX4I1 Abcam Cat# ab14744, RRID: AB_301443<br>COX5B Santa Cruz Cat# sc-374417, RRID: AB_10988066<br>COX6A1 Sigma Cat# HPA062394, RRID: AB_2684749<br>NDUFA4 Origene Cat# TA351429<br>COX14 Provided by Peter Rehling Ritcher-Dennerlein et al, 2016 2 Haga clic o pulse aquí para escribir texto.<br>HIGD2A Sigma Cat# HPA042715, RRID: AB_2678127<br>VDAC1 Abcam Cat# ab14734, RRID: AB_443084<br>TOM20 SantaCruz Cat# sc-11415, RRID: AB_2207533<br>TIMM17 Abcam Cat# ab192246<br>Anti-FLAG Abcam ab1257<br>2° Ab-mouse Rockland Immunochemicals Cat# 610-103-121 RRID: AB_218457<br>2° Ab-rabbit Rockland Immunochemicals Cat# 611-1302 RRID: AB_219720<br><br>All antibodies were used at a 1:500 dilution in the blocking solution, except for SDHA, COX1, COX5B, VDAC1, and TOM20, which were used at 1:1000, and the secondary anti-mouse and anti-rabbit antibodies, which were used at 1:10000.
Validation	Antibodies are validated by the manufacturers. No additional validation was done by the authors of this manuscript apart from immunoblotting experiments performed.

Eukaryotic cell lines

Policy information about cell lines and Sex and Gender in Research

Cell line source(s)	Flp-In TREx HEK293cell line was purchased from Thermofisher Scientific (catalog number: R78007). Human HEK293T embryonic kidney cells (CRL-3216, RRID: CVCL-0063) were purchased from ATCC. COX14::FLAG oerexpressing cell lines were generated from Flp-In TREx HEK293 cell line as described in Methods. A stable human HIGD2A knock-out (KO) line in the HEK293T background and NDUFA4KO human HEK293T cells were generated as described in Methods.
Authentication	The COX14::FLAG overexpressed cell line was validated by western blot (using FLAG Ab)
Mycoplasma contamination	Cell line tested negative for mycoplasma contamination every 6 months.
Commonly misidentified lines (See ICLAC register)	No commonly misidentified cell lines were used in the study.

Plants

Seed stocks	*Report on the source of all seed stocks or other plant material used. If applicable, state the seed stock centre and catalogue number. If plant specimens were collected from the field, describe the collection location, date and sampling procedures.*
Novel plant genotypes	*Describe the methods by which all novel plant genotypes were produced. This includes those generated by transgenic approaches, gene editing, chemical/radiation-based mutagenesis and hybridization. For transgenic lines, describe the transformation method, the number of independent lines analyzed and the generation upon which experiments were performed. For gene-edited lines, describe the editor used, the endogenous sequence targeted for editing, the targeting guide RNA sequence (if applicable) and how the editor was applied.*
Authentication	*Describe any authentication procedures for each seed stock used or novel genotype generated. Describe any experiments used to assess the effect of a mutation and, where applicable, how potential secondary effects (e.g. second site T-DNA insertions, mosiacism, off-target gene editing) were examined.*
